# Beyond the Surface: Cutaneous Vasculitis as a Sign of a Fatal Underlying Condition

**DOI:** 10.7759/cureus.91953

**Published:** 2025-09-10

**Authors:** Sirag Elaribi, Nabil Ponnambath

**Affiliations:** 1 Dermatology, Aneurin Bevan University Health Board, Newport, GBR

**Keywords:** heart failure with reduced ejection fraction, infective endocarditis, purpura, staphylococcus aureus, vasculitis

## Abstract

Cutaneous vasculitis can pose a significant diagnostic challenge due to its broad range of potential causes, including immune-mediated conditions, drug reactions, and infection-driven processes. Systemic infections can sometimes manifest initially with skin findings, including palpable purpura, petechiae, or necrotic lesions, which mimic primary vasculitic disorders. Distinguishing between infectious and non-infectious vasculitis is critical, as empirical immunosuppressive therapy in patients with an underlying infection can worsen outcomes and delay appropriate treatment. We report the case of an 81-year-old woman who presented with cutaneous vasculitis, which was ultimately found to be a manifestation of infective endocarditis affecting the aortic valve. This case highlights the need for clinicians to maintain a high index of suspicion for systemic infection, particularly infective endocarditis, in patients presenting with vasculitic skin lesions, as early recognition and appropriate antimicrobial therapy can significantly alter prognosis.

## Introduction

Cutaneous vasculitis can present as an initial symptom in a wide range of underlying medical conditions, complicating the diagnostic process, particularly when multiple potential causes are considered [1]. This diagnostic challenge carries significant risk, as empirical treatments might exacerbate the patient's condition if the true underlying cause is misdiagnosed. For instance, administering immunosuppressive therapy could be detrimental or even fatal if the vasculitis is infection-related.

The case presented here describes a patient whose initial clinical presentation included signs of cutaneous leukocytoclastic vasculitis, with an unclear underlying cause. Over time, and with further evaluation, it was determined that the vasculitis was secondary to infective endocarditis (IE). This case highlights the critical need for a thorough diagnostic approach before initiating treatment, as distinguishing between immune-mediated vasculitis and infection-driven vasculitis is essential for patient safety.

## Case presentation

An 81-year-old woman with a past medical history of atrial fibrillation, type II diabetes mellitus, hypertension, and mild left ventricular dysfunction (LVSD) was referred to the acute medical unit with a worsening purplish skin rash on her hands and lower legs that had developed over several weeks. The rash was associated with a few blisters on the feet. She also reported experiencing exertional dyspnoea and bilateral leg swelling, which began shortly before the rash appeared. She denied any fever, rigours, bleeding per orifices, or weight loss. No neurological symptoms or visual changes were reported. 

**Figure 1:**
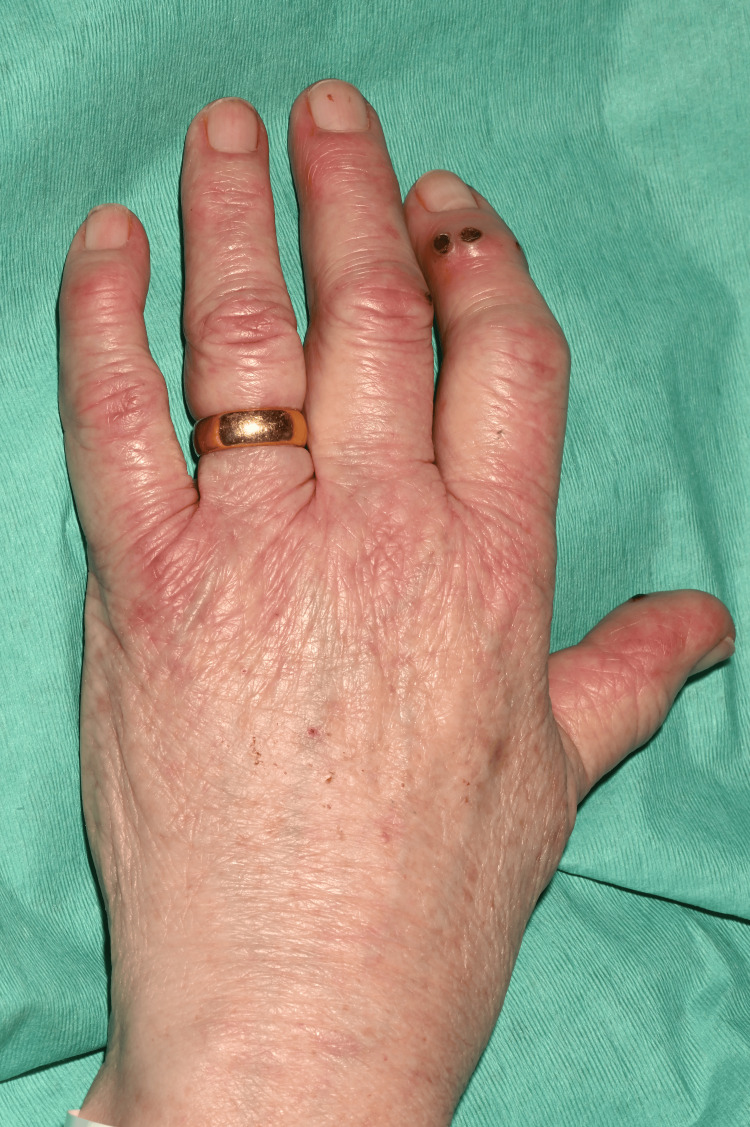
Multiple necrotic areas and superficial ulcerations on the left index.

**Figure 2:**
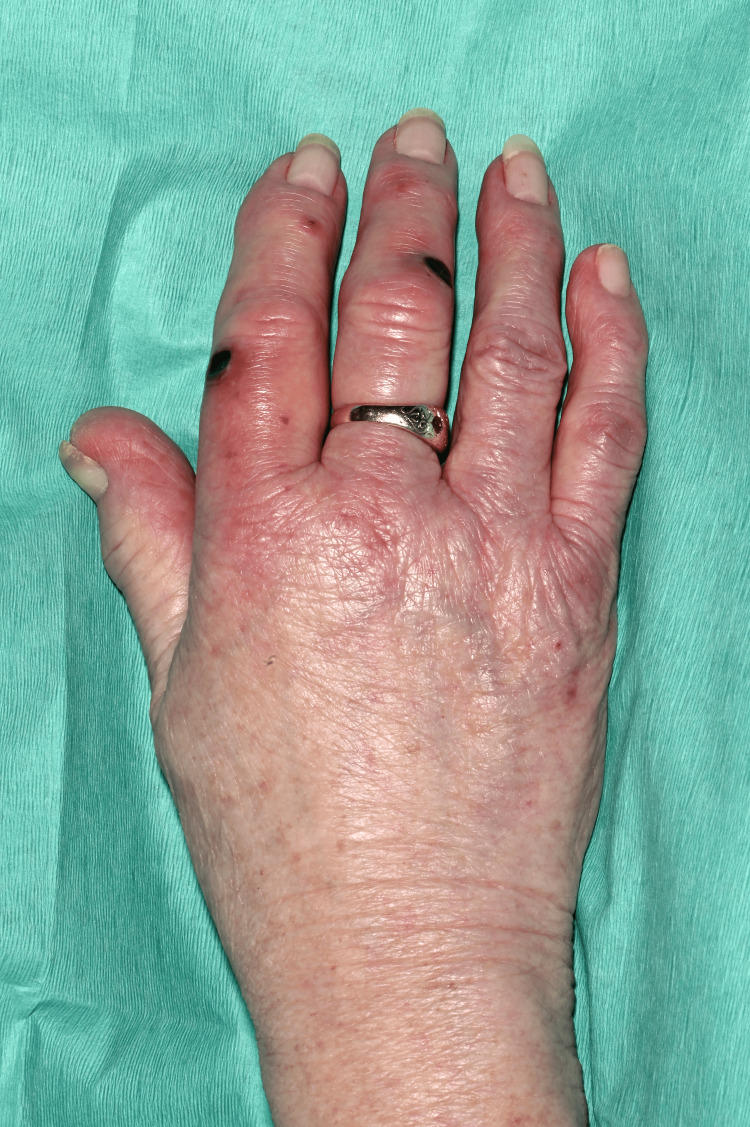
Right hand shows splinter haemorrhages, vesicles, necrosis, and superficial ulceration (index and middle finger).

**Figure 3:**
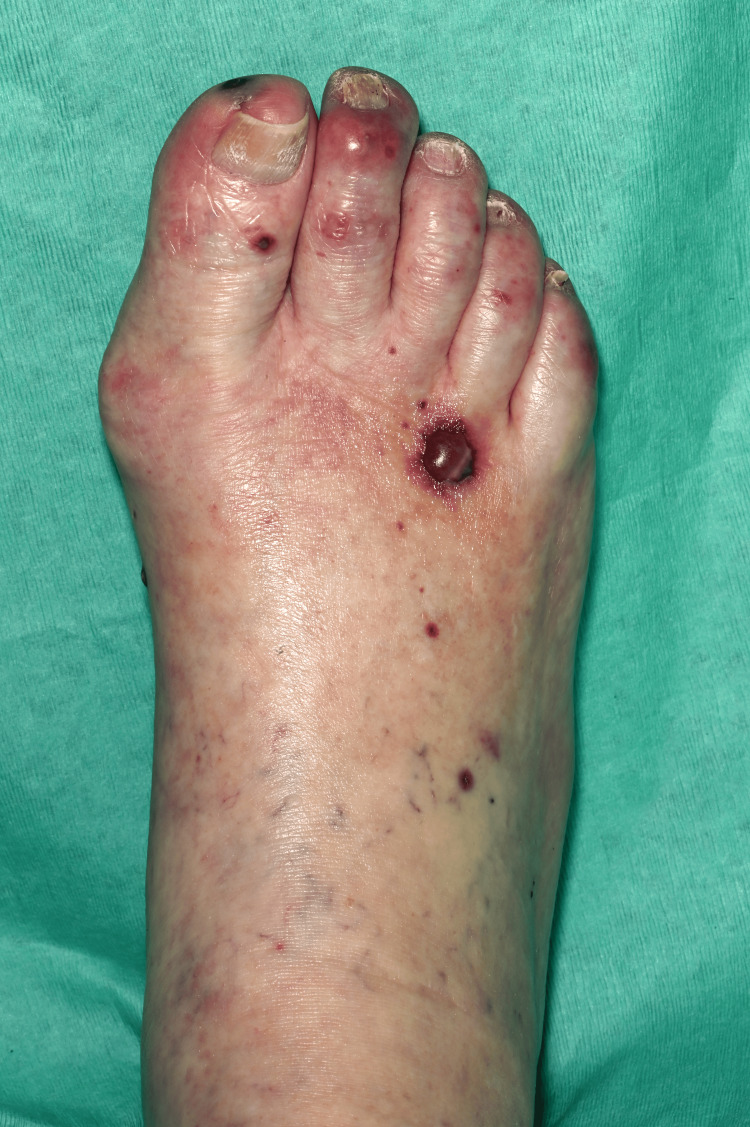
Tense blood-filled blister on the dorsum of right foot accompanied with purpura.

**Figure 4:**
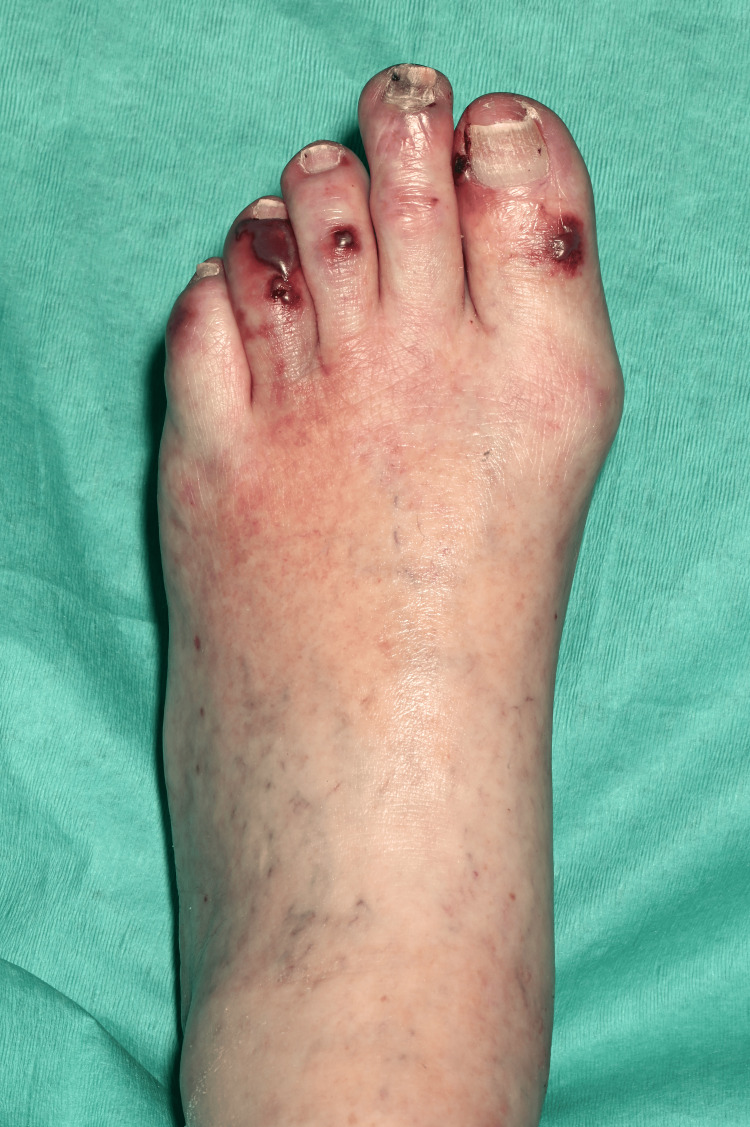
Purpura on the dorsum of left foot.

**Figure 5:**
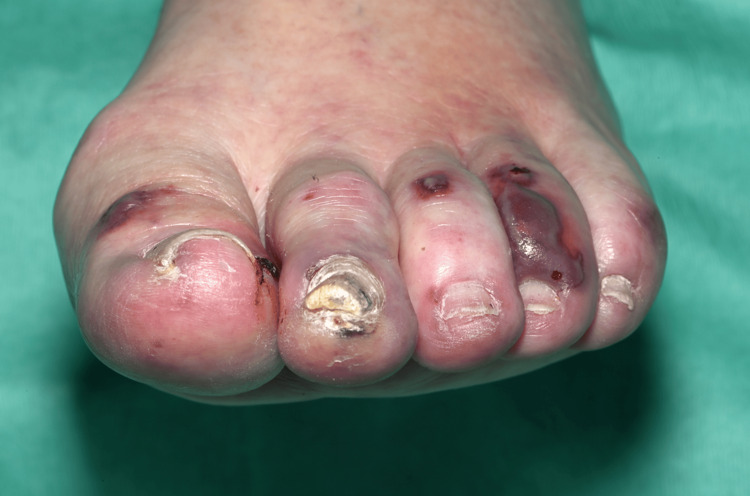
Left foot showing blood-filled blisters with splinter haemorrhages.

**Figure 6:**
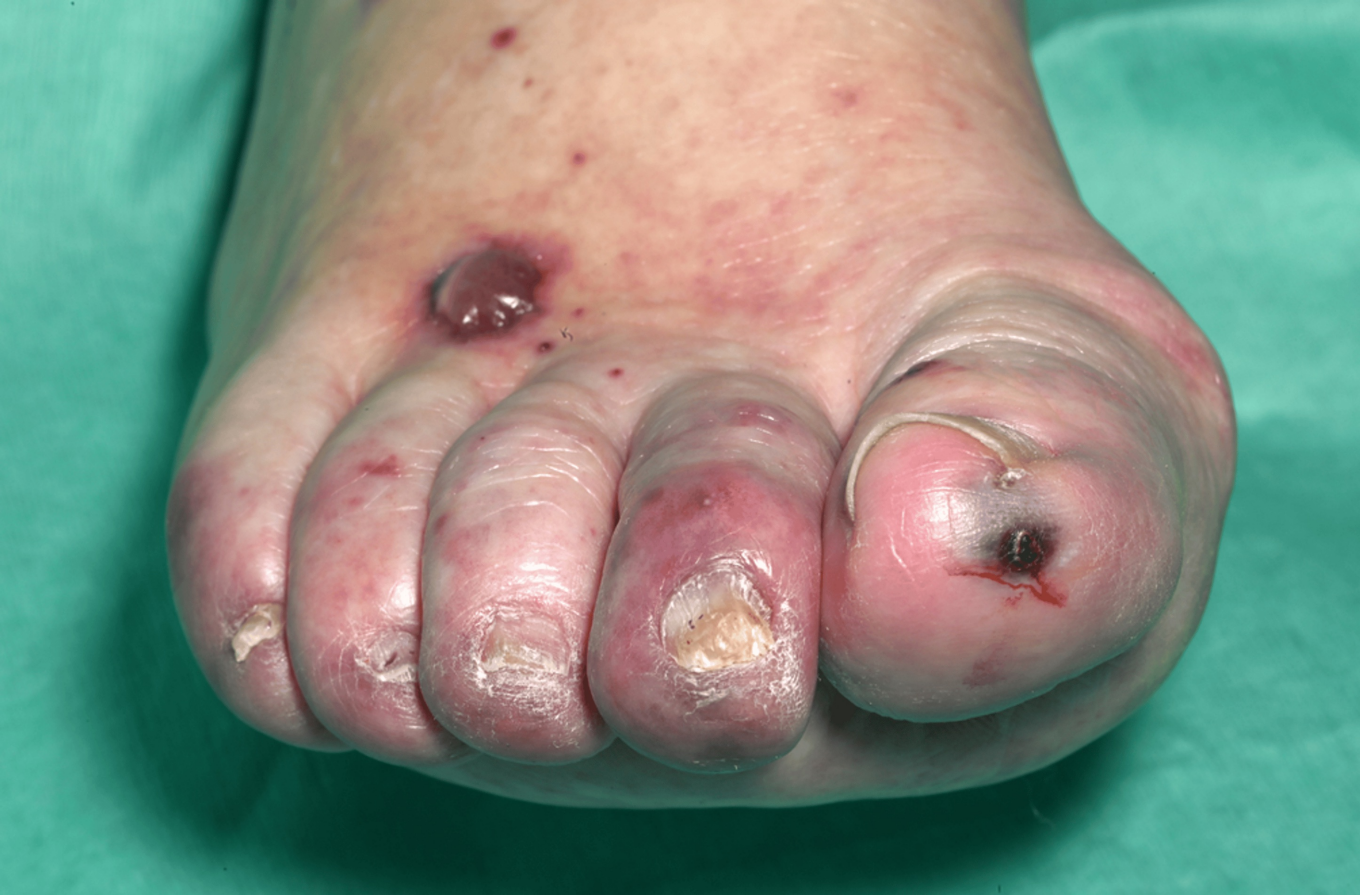
Right foot showing blood-filled blisters with splinter haemorrhages.

**Figure 7:**
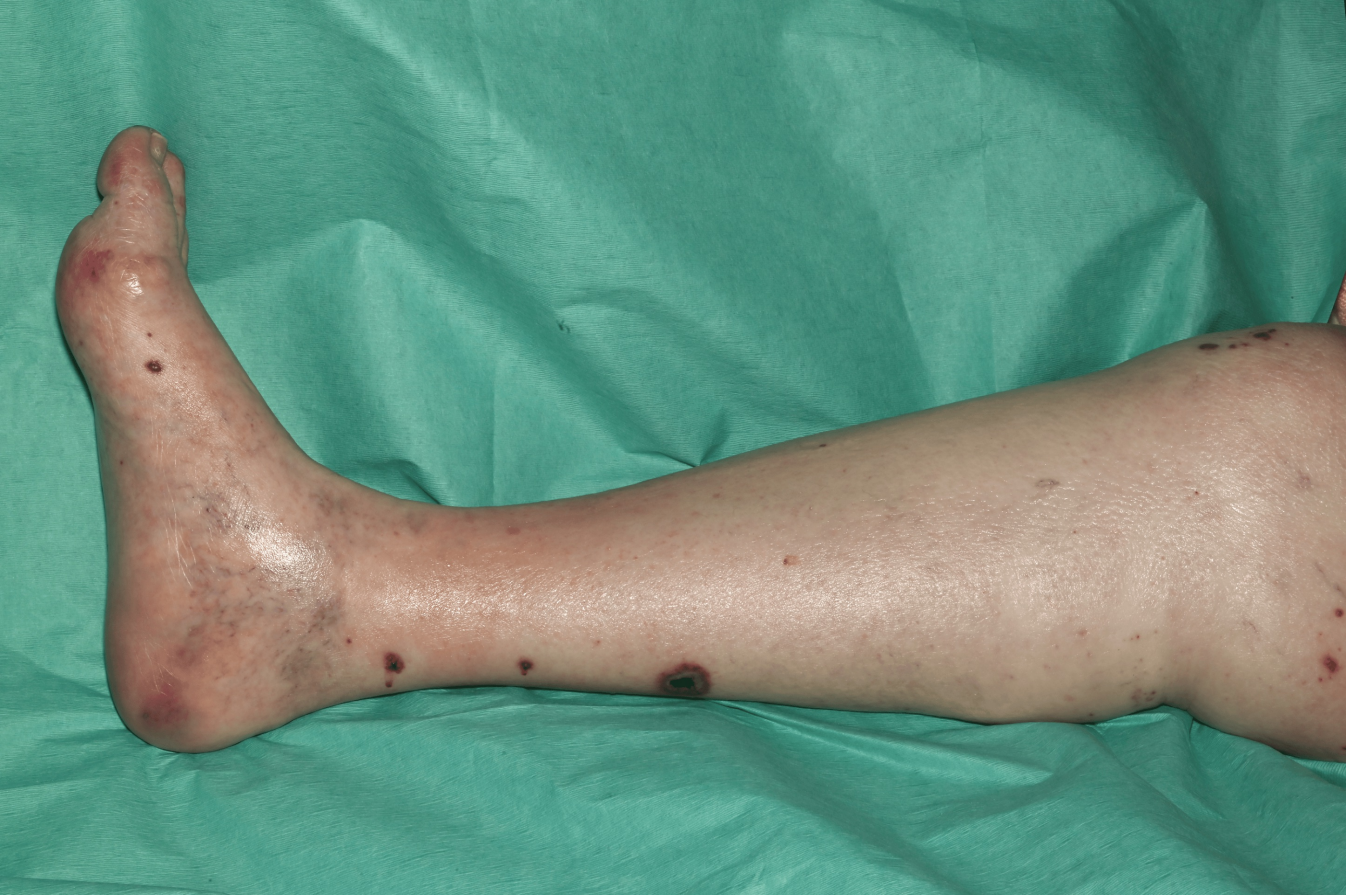
Multiple necrotic ulcers on the right leg.

**Figure 8:**
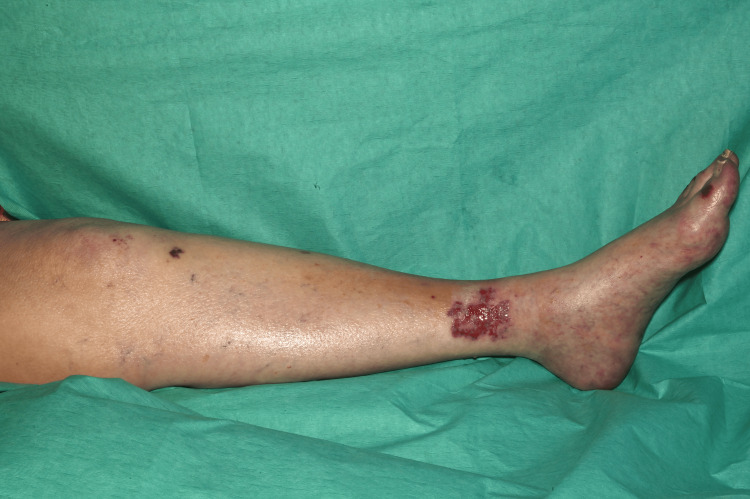
A superficial ulcer over the left medial malleolus.

**Figure 9:**
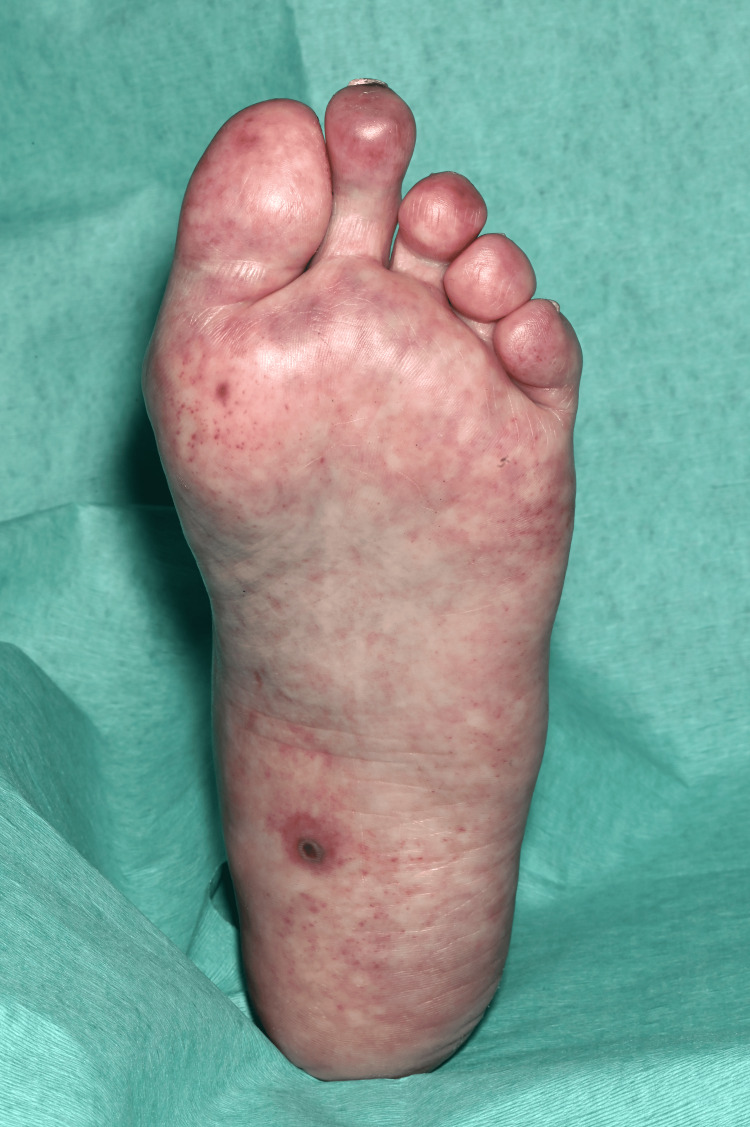
The plantar surface of the left foot shows diffuse erythematous macules and patches (Janeway lesions) with a central area of ulceration on the medial aspect of the midfoot.

**Figure 10:**
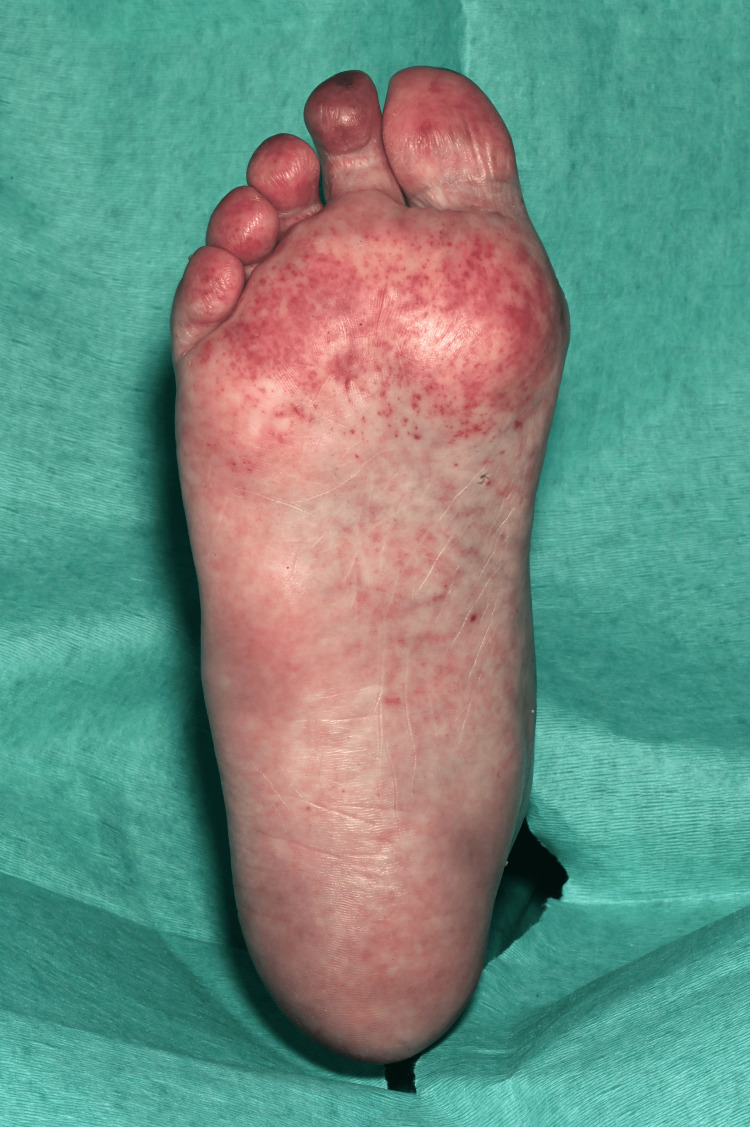
The plantar surface of the right foot shows diffuse erythematous macules and patches (Janeway lesions).

On clinical examination, she had non-blanching purpura on her hands, legs, and feet, along with a few blood-filled blisters on the feet and splinter haemorrhages (Figures 1-6). Additionally, there were multiple necrotic ulcers on her legs, including a large ulcer on the left lower leg (Figures 7-8). Haemorrhagic macules and patches were also observed in the soles (Figures 9-10). She also had pitting pedal oedema up to mid-shin and fine bibasal end-inspiratory crackles; however, no heart murmur was detected. Her vital signs were stable, and no temperature spikes were recorded.

Initial blood tests revealed mild normocytic normochromic anaemia, a normal white blood cell count, and elevated levels of C-reactive protein (CRP), procalcitonin (PCT), and alanine aminotransferase (ALT), along with mild acute kidney injury (AKI) (Table 1). The electrolyte levels and bone profile were otherwise normal. A vasculitic workup indicated positive results for anti-nuclear antibodies (ANA) and anti-neutrophil cytoplasmic antibodies (ANCA), although both myeloperoxidase (MPO) and proteinase 3 (PR3) levels were within normal ranges. Additionally, both IgG and IgA levels were mildly elevated (Table 2).

**Table 1:** Blood results on admission ALT: alanine aminotransferase; CRP: C-reactive protein; eGFR: estimated glomerular filtration rate; MCH: mean corpuscular hemoglobin; MCV: mean corpuscular volume; WBC: white blood cell count

Test	Result	Normal Range
WBC	10.8	4-11 × 10^9^/L
Haemoglobin	90	115-165 g/L
MCV	88	80-100 fL
MCH	28.3	27-35 pg
Platelets	296	150-400 × 10^9^/L
CRP	68	< 10 mg/L
Procalcitonin	0.21	< 0.05 µg/L
Sodium	133	133-146 mmol/L
Potassium	4.8	3.5-5.3 mmol/L
Urea	19.6	2.5-7.8 mmol/L
Creatinine	116	46-92 mmol/L
eGFR	39	> 90 mL/minute/1.73 m²
ALT	127	< 41 U/L

**Table 2:** Vasculitic screen ANA: antinuclear antibody; ANAPAT: antinuclear antibody pattern; ANCA: antineutrophil cytoplasmic antibody; BBV: blood-borne virus; C3: complement component 3; C4: complement component 4; dsDNA: double-stranded deoxyribonucleic acid; ENA: extractable nuclear antigen; ESR: erythrocyte sedimentation rate; MPO: myeloperoxidase; PR3: proteinase 3

Test	Result	Normal Range
ANA	Positive (titre 1:80)	-
ANAPAT	Homogenous	-
dsDNA	<9.8	< 27 IU/mL
ANCA	Positive (titre 1:40)	-
MPO	<3.2	< 20 U/mL
PR3	<2.3	< 20 U/mL
ENA	Negative	-
C3	0.96	0.75-1.65 g/L
C4	0.16	0.14-0.54 g/L
IgG	16.45	6-16 g/L
IgA	4.99	0.4-3.5 g/L
IgM	1.27	0.5-2 g/L
ESR	16	0-35 mm/hour
BBV screen	Negative	-

**Figure 11:**
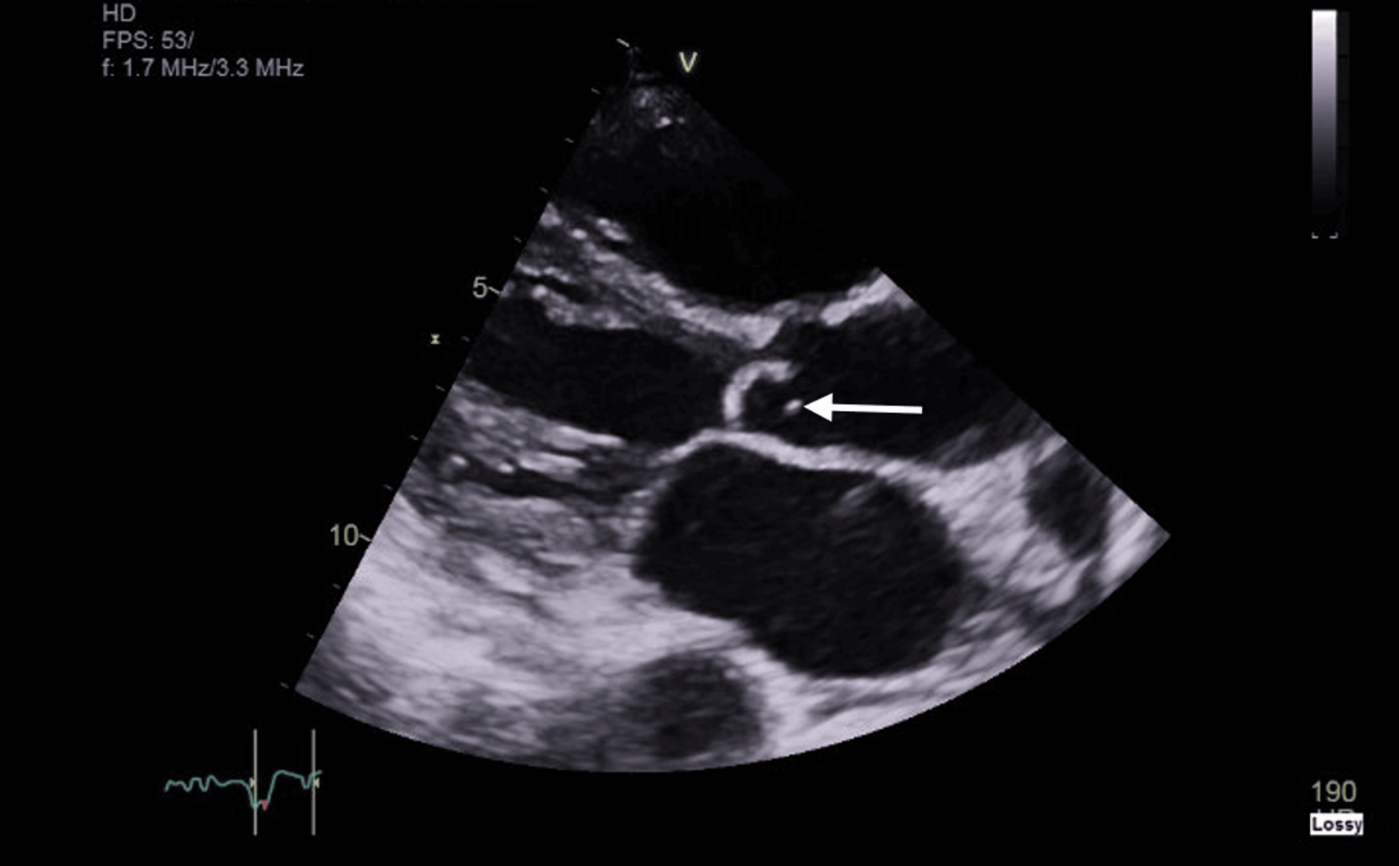
Transthoracic echocardiogram (TTE) revealing an echogenic mobile structure in the supravalvular region of the aortic valve (indicated by the arrow).

A few days later, the patient was referred to dermatology, where it was recommended to rule out IE and to consider initiating oral steroids if an infectious cause was excluded. However, over the following days, her condition worsened, with repeated blood tests showing a progressive decline in kidney function and rising CRP levels. A urine dipstick test was negative for haematuria and proteinuria. As the patient became systemically unwell and developed a fever, intravenous piperacillin/tazobactam was started to provide broad-spectrum antimicrobial coverage. Subsequently, *Staphylococcus aureus* was isolated from both the leg ulcer collected on day 1 and the peripheral blood culture obtained on day 4 of admission. This prompted the initiation of intravenous flucloxacillin and an urgent inpatient transthoracic echocardiogram (TTE) based on the advice of the microbiology team. The TTE identified an echogenic mobile structure in the supravalvular region of the aortic valve with severe left ventricular systolic dysfunction (Figure 11). The ejection fraction had declined markedly, from 39% a few weeks earlier to 10-15% at the time of the study. Ten days after admission, she was diagnosed with IE of the aortic valve and urgently transferred to a cardiology ward, where she experienced a further increase in CRP levels and worsening of her rash. 

Blood cultures taken on day 7 and day 11 of admission (two and six days after starting intravenous flucloxacillin) were both negative.

Despite receiving the appropriate antibiotic treatment, the patient's condition continued to deteriorate. After discussions with her and her family, a decision was made to transition to palliative care. She passed away on day 23 of her hospital admission, 13 days after being diagnosed with IE.

## Discussion

IE, though primarily associated with cardiac manifestations, can, on occasion, present with extracardiac symptoms such as cutaneous vasculitis. Although it is rare, there is evidence that IE can present as cutaneous vasculitis. A study conducted in 2015 explored this unusual presentation [2]. Out of 766 patients treated for cutaneous vasculitis, 27 cases were found to have bacterial infections, and six of those were confirmed to be linked to IE. The study highlighted a median delay of four days from hospital admission to IE diagnosis, but in two particularly striking cases, diagnosis was delayed by 32 and 45 days due to a lack of classic IE signs at admission. These delays were associated with adverse clinical outcomes, as both patients subsequently developed acute heart failure necessitating prosthetic valve implantation.

A case published in 2012 described a patient who presented with a vasculitic rash, anaemia, and a positive dipstick test for protein and blood. A diastolic murmur was noted on admission [3]. The initial diagnosis was Henoch-Schönlein purpura (HSP) until blood cultures taken at admission later returned positive for *Enterococcus faecalis*. Aortic valve endocarditis was confirmed by transoesophageal echocardiogram (TOE). A similar case was reported in 2017, involving a 42-year-old lady who presented with a non-blanching purpuric rash involving the lower extremities, and initial blood tests showed anaemia with raised CRP [4]. Urine dipstick was positive for blood and proteins, and the vasculitic screen was positive only for cryoglobulins. A skin biopsy showed leukocytoclastic vasculitis, and the diagnosis of HSP was made. However, immunosuppressive therapy was delayed, and antibiotics were started following positive blood cultures. TEE identified a large vegetation on the aortic valve, accompanied by severe aortic insufficiency. Notably, there were no corresponding murmurs or other abnormal cardiac findings on physical examination. In both cases, the severity of the rash improved following the aortic valve replacement, and the patients were successfully discharged from the hospital.

In 2017, a case of leukocytoclastic vasculitis associated with IE in a 31-year-old woman with transposition of the great arteries (TGA) and a prosthetic valve was reported [5]. The patient developed bacterial IE caused by *Staphylococcus aureus* after a conization procedure and presented with fever, shivering, and purpura, which was confirmed to be leukocytoclastic vasculitis through biopsy. Notably, there were no immune complex deposits or cryoglobulinemia observed in the biopsy. The patient was treated with intravenous antibiotics for six weeks, which led to the resolution of both the endocarditis and the vasculitis, without the need for immunosuppressive therapy.

Agarwal et. al reported a patient who presented with a vasculitic rash and renal failure and was initially diagnosed with essential type III cryoglobulinemia [6]. Although the patient initially responded to immunosuppressive therapy, his condition worsened six weeks later, leading to a diagnosis of subacute bacterial endocarditis. The patient showed significant improvement following antibiotic treatment.

A case of a 33-year-old man with IE complicated by ANCA-associated vasculitis (AAV) and renal failure was reported in 2020 [7]. The patient, who had a history of congenital ventricular septal defect (VSD), developed fever, joint pain, and purpura. Laboratory tests revealed elevated creatinine, PR3-ANCA, and C-ANCA levels, indicating AAV. Multiple blood cultures were positive for viridans streptococcus, and initial TTE showed no valve vegetations but VSD. However, a repeat TTE four months later revealed multiple patchy strong echoes at the right ventricular side of the septal defect, suggesting the formation of vegetations at the defect site. Furthermore, a kidney biopsy revealed necrotising glomerulonephritis consistent with ANCA-associated pauci-immune glomerulonephritis. Despite antibiotic treatment, the patient’s condition worsened, necessitating immunosuppressive therapy with corticosteroids and intravenous immunoglobulin, which led to rapid improvement.

In another case from 2021, a 71-year-old woman with a dual-chamber automatic implantable cardioverter-defibrillator (ICD) presented with septic shock, acute renal failure, and a purpuric rash with scattered bullae [8]. Initial cultures were negative, and although the biopsy confirmed leukocytoclastic vasculitis, immunosuppressive treatment was postponed due to the patient’s hemodynamic instability. Subsequent echocardiography, prompted by acute decompensation, revealed a mobile mass near the cardiac device, fulfilling a major criterion for the diagnosis of IE. The patient was treated with ceftriaxone, targeting both native and prosthetic valve endocarditis, leading to stabilisation and resolution of the vasculitis.

Two cases of cryoglobulinemic vasculitis associated with methicillin-resistant *Staphylococcus aureus* (MRSA) tricuspid valve endocarditis were reported by Josephson et. al in 2022 [9]. Both patients had a history of substance use and injection drug use. Each case involved the presence of MRSA, and both patients presented with purpuric skin lesions and positive serum cryoglobulins. Histopathology confirmed vasculitis, and both patients improved with antibiotic therapy targeting the underlying endocarditis. One patient was also treated with a short course of prednisone without adverse effects on infection control. The cases highlight the importance of considering endocarditis in patients with new cryoglobulinemic vasculitis, particularly those with risk factors such as intravenous drug use or prior endocarditis.

In 2023, a 71-year-old man with a four-month history of anaemia, thrombocytopenia, fatigue, weight loss, night sweats, and episodes of painless left-eye vision loss was admitted with exacerbated symptoms, including pedal oedema and a purpuric rash [10]. Previous investigations, including colonoscopy, endoscopy, and bone marrow biopsy, had not identified the cause. Imaging studies showed splenomegaly with splenic infarcts, and a skin biopsy confirmed leukocytoclastic vasculitis. Clinical examination revealed a pansystolic murmur at the apex and a diastolic murmur in the aortic region. Laboratory results indicated chronic anaemia, thrombocytopenia, elevated ferritin, cryoglobulins, and B2-microglobulin. TTE detected a mass on the aortic valve, prompting the initiation of empirical ceftriaxone therapy. Transoesophageal echocardiography (TOE) later confirmed the presence of valvular vegetation and severe aortic and mitral regurgitation, with blood cultures identifying *Streptococcus mitis*. While the rash improved with antibiotic treatment, the patient developed bilateral hematomas, a subarachnoid haemorrhage, and deteriorating renal function. A renal biopsy revealed postinfectious glomerulonephritis with crescent formation. Following multidisciplinary consultations, the patient underwent successful mitral and aortic valve replacement. These cases underscore the importance of recognising cutaneous manifestations of IE early, as timely diagnosis and treatment are critical for patient outcomes.

## Conclusions

IE can present with cutaneous vasculitis, though it is an uncommon manifestation that poses significant diagnostic challenges. The reviewed cases illustrate that while IE may initially mimic other conditions such as HSP or ANCA-associated vasculitis, careful and timely diagnostic evaluation is crucial. Delays in diagnosis can lead to severe complications, including heart failure and the need for surgical interventions. The variability in presentation underscores the importance of considering IE in the differential diagnosis of patients with unexplained vasculitic rashes, especially when accompanied by symptoms such as anaemia, fever, or cardiac murmurs. Early identification through appropriate imaging and blood cultures, followed by prompt treatment with antibiotics and, if necessary, surgical intervention, is vital for improving patient outcomes and preventing severe complications. The integration of dermatological, microbiological, and cardiological evaluations can enhance diagnostic accuracy and lead to more effective management of these complex cases.
